# The Tongue

**Published:** 1870-11

**Authors:** 


### THE TONGUE.

In health, the tongue has a reddish, moist appearance like the lips, extending back two or three inches.

If the tongue has a whitish coating, there is internal fever.

If the liver is torpid, is out of order, does not withdraw the bile from the blood, the man is bilious, and his tongue has a yellowish coating ; if the tongue is fiery and red, especially at the edges, there is dangerous inflammation somewhere in the system.

A black tongue indicates certain death.

A tongue natural in color, with great cracks in it, proves the presence of dyspepsia.

When the tongue has a glazed, shiny appearance, it indicates exaggerated disease ; hence the rougher the tongue is, the less glazed, the more promise there is that the health is improving.

The safest time to examine the tongue is on rising in the morning, for then there is no danger of being misled from its having been stained from eating or drinking something.

If the tongue has an unusual appearance only on one side, the presumption is that it arises from a bad tooth or other local cause.

If you feel well, never look at the tongue.

Persons in health may observe a heavy coating on the back part of the tongue in the morning; but it means nothing, for it wears off during the day.

